# Expression of Autophagy-Related Proteins in Different Types of Thyroid Cancer

**DOI:** 10.3390/ijms18030540

**Published:** 2017-03-02

**Authors:** Hye Min Kim, Eun-Sol Kim, Ja Seung Koo

**Affiliations:** Department of Pathology, Yonsei University College of Medicine, Seoul 03722, Korea; pinkmin15@yuhs.ac (H.M.K.); kesol13@yuhs.ac (E.-S.K.)

**Keywords:** autophagy, subtype, thyroid cancer, stroma, pathology

## Abstract

Thyroid cancer is common type of malignant tumor in humans, and the autophagy status of such tumors may vary according to subtype. This study aimed to investigate the expression and implications of the major autophagy-related molecules light chain (LC) 3A, LC3B, p62, and BNIP-3 in human thyroid carcinoma. Tissue microarrays were constructed from 555 thyroid cancers (papillary thyroid carcinoma (PTC): 342; follicular carcinoma (FC): 112; medullary carcinoma (MC): 70; poorly differentiated carcinoma (PDC): 23; and anaplastic carcinoma (AC): 8) and 152 follicular adenomas (FAs). Expression of autophagy-related molecules (LC3A, LC3B, p62, and BNIP-3) was detected immunohistochemically, and the results were analyzed via comparison with clinicopathologic parameters. Tumoral LC3A and LC3B expressions were the highest in MC (*p* < 0.001), whereas stromal LC3A expression was higher in AC and PTC (*p* < 0.001). BNIP-3 expression was absent in MC and AC (*p* = 0.013). Tumoral LC3A, LC3B, and p62 expressions were higher in conventional type PTC, compared with those in the follicular variant. PTC with the BRAF V600E mutation exhibited higher expression of all autophagy-related proteins relative to PTC without this mutation (*p* < 0.05). BNIP-3 negativity was associated with capsular invasion in FC (*p* = 0.001), and p62 negativity was associated with lymph node metastasis in MC (*p* = 0.006). In a univariate analysis, LC3B negativity was associated with shorter disease-free survival in PTC with the BRAF V600E mutation (*p* = 0.024). We conclude that the expression of autophagy-related proteins differs according to thyroid cancer subtype.

## 1. Introduction

Thyroid cancer is common type of malignant tumor, affecting approximately 1% of the total population. Thyroid cancer manifests as several common subtypes, including papillary thyroid carcinoma (PTC), follicular carcinoma (FC), medullary carcinoma (MC), poorly differentiated carcinoma (PDC), and anaplastic carcinoma (AC). Notably, the cellular origin, clinical manifestation, metastatic pattern, and clinical prognosis have been reported to differ depending on the subtype [1].

In the field of cancer research, autophagy, defined as the lysosomal degradation of cellular components, has recently received considerable attention. Autophagy plays an important role in homeostasis through the removal of dysfunctional or damaged cellular components and recycling of necessary cellular components [2,3,4,5]. Among the various proteins involved in the autophagy process, the following markers are commonly used to evaluate autophagic activity: light chain (LC) 3, which participates in elongation and contributes to autophagosome formation [6,7,8]; p62, a scaffold protein that transfers ubiquitinated proteins to the autophagosome [9,10]; and BCL2/adenovirus E1B 19 kDa protein-interacting protein 3 (BNIP3), a mitochondrial autophagy (mitophagy) regulator [11].

Given its role in homeostasis, autophagy acts as a double-edged sword in cancers. The high metabolic demands of highly aggressive malignant tumors cannot be satisfied by angiogenesis and/or aerobic glycolysis alone, leading to activation of the alternative metabolic pathway in which cytoplasmic components are recycled via autophagy to provide a source of cellular energy [12,13]. However, unrestrained autophagy eventually leads to cell death following the progressive consumption of cellular constituents [14,15]. Accordingly, autophagy plays roles in both tumor suppression and progression, and the autophagy status has been linked to the tumor subtype in various types of cancer [16,17,18,19,20].

In human thyroid cancer, the autophagy status may similarly vary according to the tumor subtype. However, previous studies regarding the expression of autophagy-related proteins in thyroid cancer have not yielded clear results. Therefore, in this study, we investigated the expression of LC3A, LC3B, p62, and BNIP-3, the major components of autophagy, in human thyroid carcinomas, as well as the implications of these expression patterns.

## 2. Results

### 2.1. Basal Characteristics of Thyroid Cancer

In this study, we included 555 thyroid cancer cases, including 342 cases of PTC (other subtypes: FC, 112; MC, 70; PDC, 23; and AC, 8). The basal characteristics of the PTC cases are listed in Table S1. This group comprised 302 cases of conventional type and 40 cases of follicular variant disease, and 236 of the 342 cases (69.0%) harbored the BRAF V600E mutation. The FC group comprised 99 cases of minimally invasive type and 13 cases of widely invasive type disease, for which the basal characteristics are listed in Table S2. The basal characteristics of MC, PDC, and AC are presented in Table S3.

### 2.2. Expression of Autophagy-Related Proteins in Thyroid Cancer

**Figure 1:**
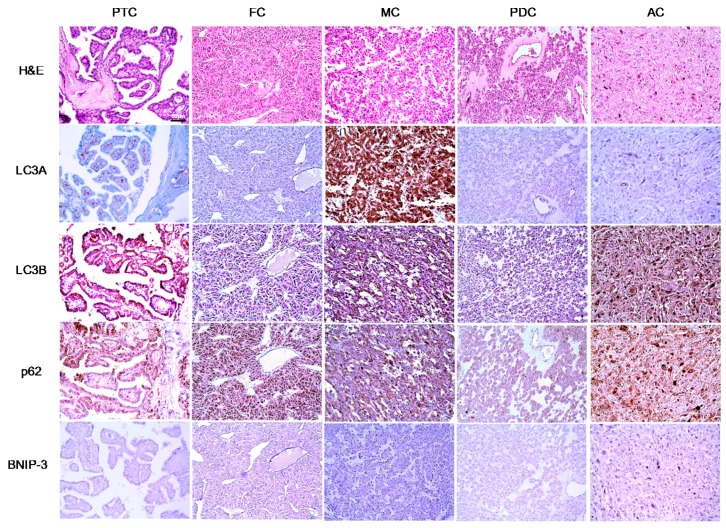
Expression of autophagy-related proteins in different types of thyroid cancer (Original magnification ×200; Scale bar, 50 μm).

We next evaluated the expression of autophagy-related proteins in thyroid cancers and observed significant differences in tumoral LC3A (*p* < 0.001), stromal LC3A (*p* < 0.001), LC3B (*p* < 0.001), and BNIP-3 (*p* = 0.016) expression patterns with respect to thyroid cancer subtype. Tumoral LC3A and LC3B expression was highest in MC, whereas stromal LC3A expression was higher in AC and PTC. BNIP-3 expression was negative in both MC and AC (Figure 1 and Table 1).

**Figure 2:**
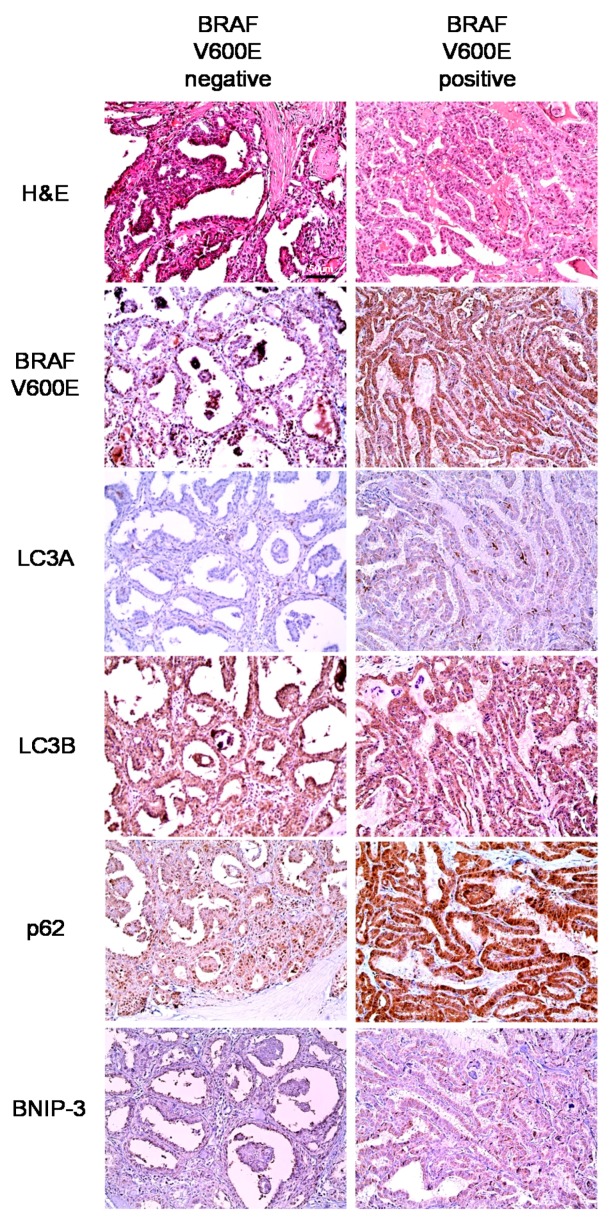
Expression of autophagy-related proteins in papillary thyroid carcinoma (PTC) according to the status of BRAF V600E mutation (Original magnification ×200; Scale bar, 50 μm).

We further evaluated the expression of autophagy-related proteins in PTCs according to the histologic subtype and BRAF V600E mutation status. We found that tumoral LC3A (*p* = 0.026), LC3B (*p* = 0.007), and p62 (*p* < 0.001) expression differed significantly according to the histologic subtype. Higher tumoral LC3A, LC3B, and p62 expressions were observed in conventional type tumors relative to follicular variant tumors. In addition, the expression of all autophagy-related proteins differed significantly according to the BRAF V600E mutation status (*p* < 0.05); specifically, the BRAF V600E mutation was associated with higher expression levels of all autophagy-related proteins (Table 2 and Figure 2). In contrast, in an analysis of follicular neoplasms, no significant differences were observed in the expression of autophagy-related proteins between FA and FC or between FC, minimally invasive and FC, widely invasive (Table 3 and Table 4).

### 2.3. Correlation Among the Expressions of Autophagy-Related Proteins

The results of the correlation analysis of the expression of LC3A, LC3B, p62, and BNIP3 showed significant correlation between LC3A (T) and LC3A (S), LC3B, p62 and BNIP3 (*r* = −0.233, 0.255, 0.132, 0.138 respectively; all *p* < 0.001); between LC3A (S) and LC3B (*r* = 0.120; *p* < 0.001); between LC3A (S) and p62 (*r* = 0.108; *p* < 0.001); between LC3B and p62 (*r* = 0.253; *p* < 0.001); between LC3B and BNIP3 (*r* = 0.058; *p* = 0.038); and between p62 and BNIP3 (*r* = 0.180; *p* < 0.001) (Table 5).

### 2.4. Correlations between Clinicopathologic Factors and Autophagy-Related Protein Expression in Thyroid Cancers

**Figure 3:**
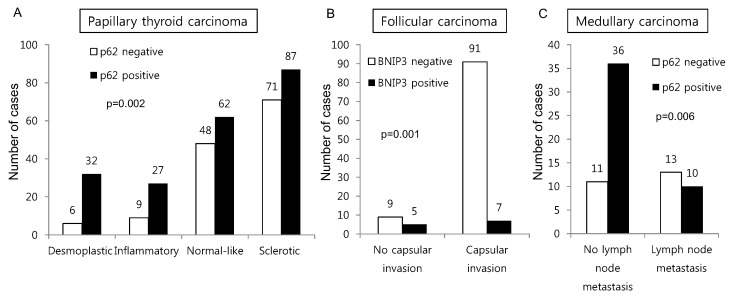
Correlations between clinicopathologic factors and autophagy-related protein expression in different types of thyroid cancer. In PTCs, p62 expression differed depending on the stromal type, whereas the proportion of tumors with positive p62 expression was higher among desmoplastic and inflammatory type tumors than among normal-like and sclerotic type tumors (**A**). BNIP-3 negativity was associated with capsular invasion in FC (**B**), and p62 negativity was associated with lymph node metastasis in MC (**C**).

Furthermore, we evaluated the correlations between clinicopathologic factors and the expression of autophagy-related proteins in thyroid cancers. In PTCs, p62 expression differed depending on the stromal type (*p* = 0.002), whereas the proportion of tumors with positive p62 expression was higher among desmoplastic and inflammatory type tumors than among normal-like and sclerotic type tumors. BNIP-3 negativity was associated with capsular invasion in FC (*p* = 0.001), and p62 negativity was associated with lymph node metastasis in MC (*p* = 0.006) (Figure 3).

### 2.5. Impact of the Expression of Autophagy-Related Proteins on the Prognosis of Patients with Thyroid Cancer

**Figure 4:**
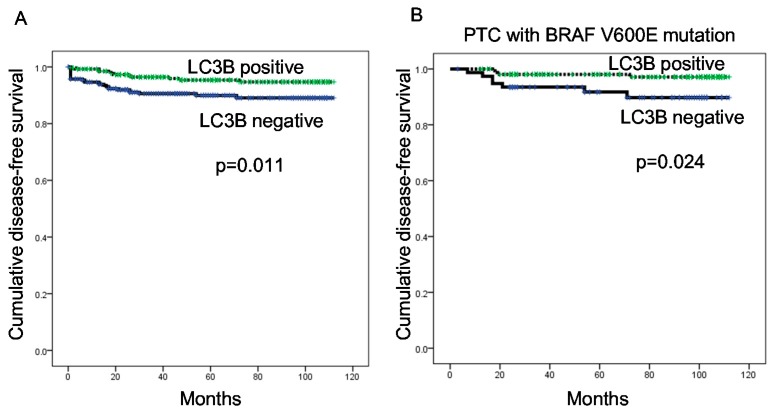
Disease-free survival among all patients with thyroid cancer (**A**); and among those with papillary thyroid carcinoma (PTC) with the V600E mutation (**B**), according to the LC3B expression status.

Finally, a logistic regression analysis was performed to evaluate the role of autophagy-related protein expression on prognosis among patients with thyroid cancer. In the univariate analysis, LC3B positivity associated with a shorter disease-free survival (*p* = 0.011). Although autophagy-related protein expression did not have a significant impact on prognosis among the total PTC group, LC3B negativity associated with a shorter disease-free survival among patients with BRAF V600E mutation-positive PTC (*p* = 0.024) (Figure 4 and Table 6).

## 3. Discussion

In this study, we investigated the expression of autophagy-related proteins in thyroid cancers and confirmed the existence of different expression patterns according to disease subtype. For example, we observed significantly higher levels of tumoral LC3A and LC3B expression in MC, a finding that was corroborated by a previous study in which higher expression levels of autophagy-related molecules, such as beclin-1 and LC3B, were observed in a MC cell line [21]. According to that earlier study, sporadic type and hereditary type MCs differed with respect to the autophagy status, and higher expression levels of autophagy-related molecules were observed in the former [21]. The different autophagy statuses observed in MC, compared to other types of thyroid cancer, might be attributable to differences in the expression of micro-RNAs (miRNAs), which have been reported to regulate autophagic activity in MC [21]. In addition, differences in miRNA expression profiles have been observed among different thyroid cancer subtypes [22]. Therefore, autophagic activity may differ as a result of distinct miRNA expression patterns, although this will require additional studies.

In our analysis of PTC, we observed differences in autophagy-related protein expression according to the BRAF V600E mutation status. Specifically, higher expression levels of all autophagy-related proteins were observed in PTCs with the BRAF V600E mutation, compared to those without the BRAF V600E mutation. In support of this finding, previous studies have observed an association between the BRAF V600E mutation and increased autophagy [23,24]. This association may be attributed to multiple factors. First, chronic ER stress may play a mechanistic role. Following activation of the IRE1/ASK1/JNK and TRB3 pathways via BRAF V600E-mediated p38 activation, Bcl-XL/Bcl-2 phosphorylation, induced by active JNK, and Akt/mTOR axis inhibition, mediated by TRB3, lead to increased autophagic activity [24]. Second, long non-coding RNAs (lncRNAs) may also play a mechanistic role. For example, an increase in the LC3-II/LC3-I that was mediated by BRAF-activated lncRNA was found to induce autophagy in a previous study [23].

Our results further demonstrated a high level of stromal LC3A expression in PTC. Previously, stromal LC3A expression was also observed in breast cancer [18], similar to the findings of this study. The expression of autophagy-related proteins in tumor stroma has been attributed to a “reverse Warburg” effect, wherein a metabolic interaction exists between breast cancer cells and stromal cells. According to this theory, glycolysis, mitochondrial dysfunction, and increased autophagy activity are induced in stromal cells by reactive oxygen species produced by breast cancer cells. Ketone bodies and lactate, the by-products of glycolysis in stromal cells, enter cancer cells and facilitate the production of ATP through oxidative phosphorylation [25,26,27]. Therefore, according to the reverse Warburg effect, autophagic activity would increase in stromal cells, which corresponds with the increased expression of LC3A and LC3B in PTC tumor stroma. In this context, cancer-associated fibroblasts that express reduced levels of caveolin-1 serve as tumor cell-interacting stromal cells [28,29]. In a previous study of thyroid cancer, the reported proportion of caveolin-1-negative stroma was 78.9% [30], suggesting that the reverse Warburg effect may describe the situation in PTC. However, further studies are needed to clarify this potential mechanism.

We note that one limitation of our study was the use of immunohistochemistry (IHC) to detect autophagy-related proteins (beclin-1, LC3A, and LC3B) as an indicator of autophagic activity. Because autophagy is a dynamic, multi-step process, a static measure of autophagy activity (e.g., IHC) may yield inaccurate results. Namely, as the autophagy-related proteins LC3A and LC3B are autophagosome components, the increased expression of these proteins could be interpreted as an increase in the number of autophagosomes and, therefore, in autophagic activity. However, an increased number of autophagosomes might also result from delayed degradation. Therefore, an analysis of autophagy flux, which indicates changes in autophagic stages, would yield a more accurate measurement of autophagic activity [10]. However, it was impossible to evaluate autophagy flux in our IHC study of paraffin-embedded tumor samples.

Clinically, the results of our study imply that autophagy regulation should be considered as a potential cancer therapeutic target. Recent evidence suggests that treatment with autophagy inhibitors could inhibit the growth of various tumors [31,32,33,34]. Specifically, autophagy inhibition might provide insights into the treatment of MC, which expresses high levels of autophagy markers and for which an effective target therapy is not currently available.

## 4. Materials and Methods

### 4.1. Patient Selection and Study Design

This study included patients with diagnosed PTC who underwent surgery at Severance Hospital between January 2012 and December 2013, as well as patients diagnosed with other thyroid cancer subtypes following surgery at Severance Hospital between January 2000 and December 2014. Patients who received preoperative chemotherapy were excluded. The study protocol was approved by the Institutional Review Board of Yonsei University Severance Hospital (8 November 2016; 4-2016-0832).

All cases of thyroid cancer were reviewed retrospectively by a single thyroid pathologist (Ja Seung Koo) via a histologic review of hematoxylin and eosin (H&E)-stained slides. Clinicopathologic data were obtained from the patients’ medical records and included the age at diagnosis, disease recurrence, metastasis status, current status, and duration of follow-up. The tumor size, location (right or left lobe), extent (confined to the thyroid parenchyma or with extrathyroidal spread), and number of metastatic lymph nodes were also determined from the histologic review of tumor slides and surgical pathology reports.

### 4.2. Tissue Microarray

Representative areas on (H&E)-stained slides were selected, and corresponding spots were marked on the surfaces of matching paraffin blocks. Three-millimeter sized tissue cores were extracted by using a manual tissue arrayer from the selected areas and placed into a 6 × 5 recipient block. More than two tissue cores were extracted from each case to minimize extraction bias. Each tissue core was assigned a unique tissue microarray location number that was linked to a database containing other clinicopathologic data.

### 4.3. Immunohistochemistry

The antibodies used for (IHC) are listed in Table 7. All IHC analyses were performed using formalin-fixed, paraffin-embedded tissue sections. Briefly, 5-μm-thick sections were obtained with a microtome, transferred onto adhesive slides, and dried at 62 °C for 30 min. After incubation with primary antibodies, immunodetection was performed using biotinylated anti-mouse immunoglobulin followed by peroxidase-labeled streptavidin, using a labeled streptavidin biotin kit with 3,3′-diaminobenzidine chromogen as the substrate. The primary antibody incubation step was omitted from the negative control. A positive control tissue was used per the manufacturer’s recommendation. Slides were counterstained with Harris hematoxylin.

### 4.4. Interpretation of Immunohistochemical Staining

Immunohistochemical marker expression was visualized using light microscopy. Stained slides were evaluated semi-quantitatively according to a previously reported method [35]. Tumor and stromal cell staining were assessed using the following scoring system: 0, negative or weak immunostaining in <1% of the tumor/stroma; 1, focal expression in 1%–10% of the tumor/stroma; 2: positive staining in 11%–50% of the tumor/stroma; and 3: positive staining in 51%–100% of the tumor/stroma. The entire tumor area was subjected to assessment, and a score of ≥2 was defined as positive. BRAF V600E staining was considered positive when more than 20% tumor cells were positive, as previously described [36].

### 4.5. Statistical Analysis

Data were analyzed using IBM SPSS Statistics for Windows, Version 21.0 (IBM Corp. Released 2012. Armonk, NY, USA). For determinations of statistical significance, Student’s *t*-test and Fisher’s exact test were used for continuous and categorical variables, respectively. The correlation between the expression of GLS1, GDH, and ASCT was analyzed using Kendall’s tau. To analyze data with multiple comparisons, a corrected *p*-value and the Bonferroni multiple comparison procedure were applied. Statistical significance was set at a *p*-value < 0.05. Kaplan–Meier survival curves and log-rank statistics were used to evaluate the time to tumor recurrence and overall survival. A Cox proportional hazards model was used for the multivariate regression analysis.

## 5. Conclusions

In conclusion, our combined data indicate that the expression of autophagy-related proteins in thyroid cancers differs according to the subtype and, among PTCs, the BRAF V600E mutation status. These findings, which corroborate the results of earlier studies, may lead to improvements and developments in the field of target therapy for thyroid cancer.

## Figures and Tables

**Table 1:** Expression of autophagy-related proteins according to histologic thyroid cancer subtype.

Parameters	Total *n *= 555 (%)	PTC *n *= 342 (%)	FC *n *= 112 (%)	MC *n *= 70 (%)	PDC *n *= 23 (%)	AC *n* = 8 (%)	*p*-Value
LC3A (T)							**<0.001**
Negative	421 (75.9)	294 (86.0)	99 (88.4)	0 (0.0)	20 (87.0)	8 (100.0)	
Positive	134 (24.1)	48 (14.0)	13 (11.6)	70 (100.0)	3 (13.0)	0 (0.0)	
LC3A (S)							**<0.001**
Negative	359 (64.7)	167 (48.8)	99 (88.4)	70 (100.0)	21 (91.3)	2 (25.0)	
Positive	196 (35.3)	175 (51.2)	13 (11.6)	0 (0.0)	2 (8.7)	6 (75.0)	
LC3B							**<0.001**
Negative	283 (51.0)	146 (42.7)	99 (88.4)	14 (20.0)	20 (87.0)	4 (50.0)	
Positive	272 (49.0)	196 (57.3)	13 (11.6)	56 (80.0)	3 (13.0)	4 (50.0)	
P62							0.220
Negative	221 (39.8)	134 (39.2)	53 (47.3)	24 (34.3)	6 (26.1)	4 (50.0)	
Positive	334 (60.2)	208 (60.8)	59 (52.7)	46 (65.7)	17 (73.9)	4 (50.0)	
BNIP-3							**0.016**
Negative	494 (89.0)	297 (86.8)	100 (89.3)	70 (100.0)	19 (82.6)	8 (100.0)	
Positive	61 (11.0)	45 (13.2)	12 (10.7)	0 (0.0)	4 (17.4)	0 (0.0)	

PTC: papillary thyroid carcinoma; FC: follicular carcinoma; MC: medullary carcinoma; PDC: poorly differentiated carcinoma; AC: anaplastic carcinoma; T: tumoral; S: stromal. Bold indicates statistically significant (*p* < 0.05).

**Table 2:** Expression of autophagy-related proteins according to histologic papillary thyroid carcinoma subtype.

Parameters	Total *n* = 342 (%)	Histologic Subtype		*p*-Value	BRAF V600E Mutation Status		*p*-Value
		Conventional Type *n* = 302 (%)	Follicular Variant *n* = 40 (%)		No Mutation *n* = 106 (%)	Mutation *n* = 236 (%)	
LC3A (T)				**0.026**			**0.002**
Negative	294 (86.0)	255 (84.4)	39 (97.5)		100 (94.3)	194 (82.2)	
Positive	48 (14.0)	47 (15.6)	1 (2.5)		6 (5.7)	42 (17.8)	
LC3A (S)				0.133			**0.031**
Negative	167 (48.8)	143 (47.4)	24 (60.0)		61 (57.5)	106 (44.9)	
Positive	175 (51.2)	159 (52.6)	16 (40.0)		45 (42.5)	130 (55.1)	
LC3B				**0.007**			**<0.001**
Negative	146 (42.7)	121 (40.1)	25 (62.5)		68 (64.2)	78 (33.1)	
Positive	196 (57.3)	181 (59.9)	15 (37.5)		38 (35.8)	158 (66.9)	
p62				**<0.001**			**<0.001**
Negative	134 (39.2)	105 (34.8)	29 (72.5)		72 (67.9)	62 (26.3)	
Positive	208 (60.8)	197 (65.2)	11 (27.5)		34 (32.1)	174 (73.7)	
BNIP-3				0.327			**<0.001**
Negative	297 (86.8)	260 (86.1)	37 (92.5)		105 (99.1)	192 (81.4)	
Positive	45 (13.2)	42 (13.9)	3 (7.5)		1 (0.9)	44 (18.6)	

T: tumoral; S: stromal. Bold indicates statistically significant (*p* < 0.05).

**Table 3:** Expression of autophagy-related proteins in follicular neoplasms.

Parameters	Total *n* = 265 (%)	FA *n* = 153 (%)	FC *n* = 112 (%)	*p*-Value
LC3A (T)				0.300
Negative	240 (90.6)	141 (92.2)	99 (88.4)	
Positive	25 (9.4)	12 (7.8)	13 (11.6)	
LC3A (S)				0.278
Negative	227 (85.7)	128 (83.7)	99 (88.4)	
Positive	38 (14.3)	25 (16.3)	13 (11.6)	
LC3B				0.221
Negative	226 (85.3)	127 (83.0)	99 (88.4)	
Positive	39 (14.7)	26 (17.0)	13 (11.6)	
p62				0.185
Negative	138 (52.1)	85 (55.6)	53 (47.3)	
Positive	127 (47.9)	68 (44.4)	59 (52.7)	
BNIP-3				0.946
Negative	237 (89.7)	137 (89.5)	100 (89.3)	
Positive	28 (10.6)	16 (10.5)	12 (10.7)	

FA: follicular adenoma; FC: follicular carcinoma; T: tumoral; S: stromal.

**Table 4:** Expression of autophagy-related proteins according to histologic follicular carcinoma (FC) subtype.

Parameters	Total *n* = 112 (%)	FC, Minimally Invasive Type *n* = 99 (%)	FC, Widely Invasive Type *n* = 13 (%)	*p*-Value
LC3A (T)				0.646
Negative	99 (88.4)	88 (88.9)	11 (84.6)	
Positive	13 (11.6)	11 (11.1)	2 (15.4)	
LC3A (S)				1.000
Negative	99 (88.4)	87 (87.9)	12 (92.3)	
Positive	13 (11.6)	12 (12.1)	1 (7.7)	
LC3B				0.651
Negative	99 (88.4)	11 (84.6)	88 (88.9)	
Positive	13 (11.6)	2 (15.4)	11 (11.1)	
p62				0.616
Negative	53 (47.3)	46 (46.5)	7 (53.8)	
Positive	59 (52.7)	53 (53.5)	6 (46.2)	
BNIP-3				0.354
Negative	100 (89.3)	87 (87.9)	13 (100.0)	
Positive	12 (10.7)	12 (12.1)	0 (0.0)	

T: tumoral; S: stromal.

**Table 5:** Correlation among the expressions of autophagy-related proteins.

Parameters	LC3A (T)	LC3A (S)	LC3B	p62
LC3A (T)				
Correlation coefficient	-	-	-	-
*p*-value	-	-	-	-
LC3A (S)				
Correlation coefficient	−0.233	-	-	-
*p*-value	**<0.001**	-	-	-
LC3B				
Correlation coefficient	0.255	0.120	-	-
*p*-value	**<0.001**	**<0.001**	-	-
p62				
Correlation coefficient	0.132	0.108	0.253	-
*p*-value	**<0.001**	**<0.001**	**<0.001**	-
BNIP3				
Correlation coefficient	0.138	0.029	0.058	0.180
*p*-value	**<0.001**	0.297	**0.038**	**<0.001**

T: tumoral; S: stromal. Bold indicates statistically significant (*p* < 0.05).

**Table 6:** Univariate analysis of the influences of autophagy-related protein expression on disease-free and overall survival among patients with papillary thyroid cancer (log-rank test).

Parameter	Number of Patients /Recurrence/Death	Disease-Free Survival		Overall Survival	
		Mean Survival (95% CI) Months	*p*-Value	Mean Survival (95% CI) Months	*p*-Value
LC3A (T)			0.717		0.327
Negative	294/16/14	106 (104–109)		108 (106–110)	
Positive	48/2/4	106 (100–111)		103 (97–109)	
LC3A (S)			0.923		0.216
Negative	167/9/6	107 (103–110)		109 (107–111)	
Positive	175/9/12	105 (102–108)		104 (101–107)	
LC3B			0.257		0.805
Negative	146/10/7	105 (101–109)		108 (105–111)	
Positive	196/8/11	108 (105–110)		107 (105–110)	
p62			0.643		0.140
Negative	134/8/4	105 (101–109)		108 (106–111)	
Positive	208/10/14	107 (104–110)		106 (103–109)	
BNIP-3			0.804		0.055
Negative	297/16/13	106 (104–109)		108 (106–110)	
Positive	45/2/5	107 (101–113)		102 (95–110)	

CI: confidence interval; T: tumoral; S: stromal.

**Table 7:** Sources, clones, and dilutions of antibodies used for immunohistochemistry.

Antibody	Clone	Dilution	Company
LC3A	EP1528Y	1:100	Abcam, Cambridge, UK
LC3B	Polyclonal	1:100	Abcam, Cambridge, UK
p62	SQSTM1	1:100	Abcam, Cambridge, UK
BNIP-3	Ana40	1:100	Abcam, Cambridge, UK
BRAF V600E	VE1	1:50	Ventana, Tucson, AZ, USA

## Supplementary Materials

Supplementary materials can be found at www.mdpi.com/1422-0067/18/3/540/s1.

## References

[1] Sherman S.I. (2003). Thyroid carcinoma. Lancet.

[2] Levine B., Klionsky D.J. (2004). Development by self-digestion: Molecular mechanisms and biological functions of autophagy. Dev. Cell.

[3] Mizushima N. (2007). Autophagy: Process and function. Genes Dev..

[4] Mizushima N., Levine B., Cuervo A.M., Klionsky D.J. (2008). Autophagy fights disease through cellular self-digestion. Nature.

[5] Yang Z., Klionsky D.J. (2010). Eaten alive: A history of macroautophagy. Nat. Cell Biol..

[6] Kabeya Y., Mizushima N., Ueno T., Yamamoto A., Kirisako T., Noda T., Kominami E., Ohsumi Y., Yoshimori T. (2000). LC3, a mammalian homologue of yeast Apg8p, is localized in autophagosome membranes after processing. EMBO J..

[7] Sivridis E., Koukourakis M.I., Zois C.E., Ledaki I., Ferguson D.J., Harris A.L., Gatter K.C., Giatromanolaki A. (2010). LC3A-positive light microscopy detected patterns of autophagy and prognosis in operable breast carcinomas. Am. J. Pathol..

[8] Yoshioka A., Miyata H., Doki Y., Yamasaki M., Sohma I., Gotoh K., Takiguchi S., Fujiwara Y., Uchiyama Y., Monden M. (2008). LC3, an autophagosome marker, is highly expressed in gastrointestinal cancers. Int. J. Oncol..

[9] Komatsu M., Waguri S., Koike M., Sou Y.S., Ueno T., Hara T., Mizushima N., Iwata J., Ezaki J., Murata S. (2007). Homeostatic levels of p62 control cytoplasmic inclusion body formation in autophagy-deficient mice. Cell.

[10] Mizushima N., Yoshimori T., Levine B. (2010). Methods in mammalian autophagy research. Cell.

[11] Quinsay M.N., Thomas R.L., Lee Y., Gustafsson A.B. (2010). BNIP3-mediated mitochondrial autophagy is independent of the mitochondrial permeability transition pore. Autophagy.

[12] Degenhardt K., Mathew R., Beaudoin B., Bray K., Anderson D., Chen G., Mukherjee C., Shi Y., Gelinas C., Fan Y. (2006). Autophagy promotes tumor cell survival and restricts necrosis, inflammation, and tumorigenesis. Cancer Cell.

[13] Roy S., Debnath J. (2010). Autophagy and tumorigenesis. Semin. Immunopathol..

[14] Baehrecke E.H. (2005). Autophagy: Dual roles in life and death?. Nat. Rev. Mol. Cell Biol..

[15] Debnath J., Baehrecke E.H., Kroemer G. (2005). Does autophagy contribute to cell death?. Autophagy.

[16] Costa J.R., Prak K., Aldous S., Gewinner C.A., Ketteler R. (2016). Autophagy gene expression profiling identifies a defective microtubule-associated protein light chain 3A mutant in cancer. Oncotarget.

[17] Cha Y.J., Kim Y.H., Cho N.H., Koo J.S. (2014). Expression of autophagy related proteins in invasive lobular carcinoma: Comparison to invasive ductal carcinoma. Int. J. Clin. Exp. Pathol..

[18] Choi J., Jung W., Koo J.S. (2013). Expression of autophagy-related markers beclin-1, light chain 3A, light chain 3B and p62 according to the molecular subtype of breast cancer. Histopathology.

[19] Kim S., Jung W.H., Koo J.S. (2012). Differences in autophagy-related activity by molecular subtype in triple-negative breast cancer. Tumour Biol. J. Int. Soc. Oncodev. Biol. Med..

[20] Kim S.K., Jung W.H., Koo J.S. (2013). Expression of autophagy-related proteins in phyllodes tumor. Int. J. Clin. Exp. Pathol..

[21] Gundara J.S., Zhao J., Gill A.J., Lee J.C., Delbridge L., Robinson B.G., McLean C., Serpell J., Sidhu S.B. (2015). Noncoding RNA blockade of autophagy is therapeutic in medullary thyroid cancer. Cancer Med..

[22] Hu Y., Wang H., Chen E., Xu Z., Chen B., Lu G. (2016). Candidate microRNAs as biomarkers of thyroid carcinoma: A systematic review, meta-analysis, and experimental validation. Cancer Med..

[23] Wang Y., Guo Q., Zhao Y., Chen J., Wang S., Hu J., Sun Y. (2014). BRAF-activated long non-coding RNA contributes to cell proliferation and activates autophagy in papillary thyroid carcinoma. Oncol. Lett..

[24] Corazzari M., Rapino F., Ciccosanti F., Giglio P., Antonioli M., Conti B., Fimia G.M., Lovat P.E., Piacentini M. (2015). Oncogenic BRAF induces chronic ER stress condition resulting in increased basal autophagy and apoptotic resistance of cutaneous melanoma. Cell Death Differ..

[25] Pavlides S., Whitaker-Menezes D., Castello-Cros R., Flomenberg N., Witkiewicz A.K., Frank P.G., Casimiro M.C., Wang C., Fortina P., Addya S. (2009). The reverse Warburg effect: Aerobic glycolysis in cancer associated fibroblasts and the tumor stroma. Cell Cycle.

[26] Bonuccelli G., Tsirigos A., Whitaker-Menezes D., Pavlides S., Pestell R.G., Chiavarina B., Frank P.G., Flomenberg N., Howell A., Martinez-Outschoorn U.E. (2010). Ketones and lactate “fuel” tumor growth and metastasis: Evidence that epithelial cancer cells use oxidative mitochondrial metabolism. Cell Cycle.

[27] Martinez-Outschoorn U.E., Balliet R.M., Rivadeneira D.B., Chiavarina B., Pavlides S., Wang C., Whitaker-Menezes D., Daumer K.M., Lin Z., Witkiewicz A.K. (2010). Oxidative stress in cancer associated fibroblasts drives tumor-stroma co-evolution: A new paradigm for understanding tumor metabolism, the field effect and genomic instability in cancer cells. Cell Cycle.

[28] Sotgia F., Del Galdo F., Casimiro M.C., Bonuccelli G., Mercier I., Whitaker-Menezes D., Daumer K.M., Zhou J., Wang C., Katiyar S. (2009). Caveolin-1^−/−^ null mammary stromal fibroblasts share characteristics with human breast cancer-associated fibroblasts. Am. J. Pathol..

[29] Pavlides S., Tsirigos A., Vera I., Flomenberg N., Frank P.G., Casimiro M.C., Wang C., Fortina P., Addya S., Pestell R.G. (2010). Loss of stromal caveolin-1 leads to oxidative stress, mimics hypoxia and drives inflammation in the tumor microenvironment, conferring the “reverse warburg effect”: A transcriptional informatics analysis with validation. Cell Cycle.

[30] Kim D., Kim H., Koo J.S. (2012). Expression of caveolin-1, caveolin-2 and caveolin-3 in thyroid cancer and stroma. Pathobiol. J. Immunopathol. Mol. Cell. Biol..

[31] Amaravadi R.K., Yu D., Lum J.J., Bui T., Christophorou M.A., Evan G.I., Thomas-Tikhonenko A., Thompson C.B. (2007). Autophagy inhibition enhances therapy-induced apoptosis in a Myc-induced model of lymphoma. J. Clin. Investig..

[32] Carew J.S., Medina E.C., Esquivel J.A., Mahalingam D., Swords R., Kelly K., Zhang H., Huang P., Mita A.C., Mita M.M. (2010). Autophagy inhibition enhances vorinostat-induced apoptosis via ubiquitinated protein accumulation. J. Cell. Mol. Med..

[33] Carew J.S., Nawrocki S.T., Kahue C.N., Zhang H., Yang C., Chung L., Houghton J.A., Huang P., Giles F.J., Cleveland J.L. (2007). Targeting autophagy augments the anticancer activity of the histone deacetylase inhibitor SAHA to overcome Bcr-Abl-mediated drug resistance. Blood.

[34] Gupta A., Roy S., Lazar A.J., Wang W.L., McAuliffe J.C., Reynoso D., McMahon J., Taguchi T., Floris G., Debiec-Rychter M. (2010). Autophagy inhibition and antimalarials promote cell death in gastrointestinal stromal tumor (gist). Proc. Natl. Acad. Sci. USA.

[35] Henry L.R., Lee H.O., Lee J.S., Klein-Szanto A., Watts P., Ross E.A., Chen W.T., Cheng J.D. (2007). Clinical implications of fibroblast activation protein in patients with colon cancer. Clin. Cancer Res. Off. J. Am. Assoc. Cancer Res..

[36] Bullock M., O’Neill C., Chou A., Clarkson A., Dodds T., Toon C., Sywak M., Sidhu S.B., Delbridge L.W., Robinson B.G. (2012). Utilization of a MAB for BRAF^V600E^ detection in papillary thyroid carcinoma. Endocr. Relat. Cancer.

