# Supplementary material for: Expression of Autophagy-Related Proteins in Different Types of Thyroid Cancer
Source: Int J Mol Sci. 2017 Mar 2;18(3):540. doi: 10.3390/ijms18030540 (PMC5372556; doi:10.3390/ijms18030540)
Supplement: Supplementary file 1 [file ijms-18-00540-s001.pdf]

Supplementary Materials: Expression of Autophagy-Related Proteins in Thyroid Cancer

Hye Min Kim, Eun-Sol Kim, Ja Seung Koo

Table S1. Basal characteristics of thyroid papillary carcinoma.

Parameters	Total *n* = 342 (%)	Histologic Subtype		*p*-Value	BRAF V600E Mutation Status		*p*-Value
		Conventional Type *n* = 302 (%)	Follicular Variant *n* = 40 (%)		No Mutation *n* = 106 (%)	Mutation *n* = 236 (%)	
Age (years)				0.738			0.088
<45	154 (45.0)	135 (44.7)	19 (47.5)		55 (51.9)	99 (41.9)	
≥45	188 (55.0)	167 (55.3)	21 (52.5)		51 (48.1)	137 (58.1)	
Sex				0.945			0.212
Male	67 (19.6)	59 (19.5)	8 (20.0)		25 (23.6)	42 (17.8)	
Female	275 (80.4)	243 (80.5)	32 (80.0)		81 (76.4)	194 (82.2)	
Tumor size (cm)				0.948			0.527
≤2.0	270 (78.9)	238 (78.8)	32 (80.0)		80 (75.5)	190 (80.5)	
>2.0, ≤4.0	65 (19.0)	58 (19.2)	7 (17.5)		23 (21.7)	42 (17.8)	
>4.0	7 (2.0)	6 (2.0)	1 (2.5)		3 (2.8)	4 (1.7)	
Tumor margin				**0.002**			**0.004**
Infiltrative	287 (83.9)	260 (86.1)	27 (67.5)		80 (75.5)	207 (87.7)	
Expanding	55 (16.1)	42 (13.9)	13 (32.5)		26 (24.5)	29 (12.3)	
Tumor extension				0.344			0.193
Intrathyroidal	106 (31.0)	91 (30.1)	15 (37.5)		38 (35.8)	68 (28.8)	
Extrathyroidal	236 (69.0)	211 (69.9)	25 (62.5)		68 (64.2)	168 (71.2)	
Histologic subtype							**<0.001**
Conventional					81 (76.4)	221 (93.6)	
Follicular					25 (23.6)	15 (6.4)	
LN metastasis				0.186			0.085
No	138 (40.4)	118 (39.1)	20 (50.0)		50 (47.2)	88 (37.3)	
Yes	204 (59.6)	184 (60.9)	20 (50.0)		56 (52.8)	148 (62.7)	
Distant metastasis				0.937			0.457
No	324 (94.7)	286 (94.7)	38 (95.0)		99 (93.4)	225 (95.3)	
Yes	18 (5.3)	16 (5.3)	2 (5.0)		7 (6.6)	11 (4.7)	
Treatment				0.153			0.063
No treatment	74 (21.6)	62 (20.5)	12 (30.0)		29 (27.4)	45 (19.1)	
I-131 therapy	266 (77.8)	238 (78.8)	28 (70.0)		77 (72.6)	189 (80.1)	
Chemotherapy	0 (0.0)	0 (0.0)	0 (0.0)		0 (0.0)	0 (0.0)	
Radiotherapy	2 (0.6)	2 (0.7)	0 (0.0)		0 (0.0)	2 (0.8)	

Bold indicates statistically significant ($p < 0.05$).

Table S2. Basal characteristics of thyroid follicular carcinoma.

Parameters	Total *n* = 112 (%)	FC, Minimally Invasive Type *n* = 99 (%)	FC, Widely Invasive Type *n* = 13 (%)	*p*-Value
Age (years)				0.255
<45	51 (45.5)	47 (47.5)	4 (30.8)	
≥45	61 (54.5)	52 (52.5)	9 (69.2)	
Sex				0.233
Male	28 (25.0)	23 (23.2)	5 (38.5)	
Female	84 (75.0)	76 (76.8)	8 (61.5)	
Tumor size (cm)				**0.040**
≤2.0	34 (30.4)	34 (34.3)	0 (0.0)	
>2.0, ≤4.0	49 (43.8)	41 (41.4)	8 (61.5)	
>4.0	29 (25.9)	24 (24.2)	5 (38.5)	
Capsular invasion				0.147
No	14 (12.5)	14 (14.1)	0 (0.0)	
Yes	98 (87.5)	85 (85.9)	13 (100.0)	
Vascular invasion				**0.028**
No	66 (58.9)	62 (62.6)	4 (30.8)	
Yes	46 (41.1)	37 (37.4)	9 (69.2)	
Tumor extension				**<0.001**
Intrathyroidal	95 (84.8)	89 (89.9)	6 (46.2)	
Extrathyroidal	17 (15.2)	10 (10.1)	7 (53.8)	
LN metastasis				0.220
No	110 (98.2)	98 (99.0)	12 (92.3)	
Yes	2 (1.8)	1 (1.0)	1 (7.7)	
Distant metastasis				**0.003**
No	101 (90.2)	93 (93.9)	8 (61.5)	
Yes	11 (9.8)	6 (6.1)	5 (38.5)	
Treatment				**<0.001**
No treatment	66 (58.9)	66 (66.7)	0 (0.0)	
I-131 therapy	35 (31.3)	28 (28.3)	7 (53.8)	
Chemotherapy	1 (0.9)	0 (0.0)	1 (7.7)	
Radiotherapy	10 (8.9)	5 (5.1)	5 (38.5)	

FC: follicular carcinoma. Bold indicates statistically significant ($p < 0.05$).

Table S3. Basal characteristics of thyroid medullary carcinoma, poorly differentiated carcinoma and anaplastic carcinoma.

Parameters	MC, *n* = 70 (%)	PDC, *n* = 23 (%)	AC, *n* = 8 (%)
Age (years)			
<45	21 (30.0)	4 (17.4)	0 (0.0)
≥45	49 (70.0)	19 (82.6)	8 (100.0)
Sex			
Male	22 (31.4)	10 (43.5)	1 (12.5)
Female	48 (68.6)	13 (56.5)	7 (87.5)
Tumor size (cm)			
≤2.0	53 (75.7)	8 (34.8)	0 (0.0)
>2.0, ≤4.0	14 (20.0)	9 (39.1)	1 (12.5)
>4.0	3 (4.3)	6 (26.1)	7 (87.5)
Tumor margin			
Infiltrative	45 (64.3)	17 (73.9)	8 (100.0)
Expanding	25 (35.7)	6 (26.1)	0 (0.0)
Tumor extension			
Intrathyroidal	52 (74.3)	11 (47.8)	0 (0.0)
Extrathyroidal	18 (25.7)	12 (52.2)	8 (100.0)
LN metastasis			
No	47 (67.1)	22 (95.7)	4 (50.0)
Yes	23 (32.9)	1 (4.3)	4 (50.0)
Distant metastasis			
No	67 (95.7)	16 (69.6)	8 (100.0)
Yes	3 (4.3)	7 (30.4)	0 (0.0)
Treatment			
No treatment	61 (87.1)	7 (30.4)	2 (25.0)
I-131 therapy	4 (5.7)	9 (39.1)	0 (0.0)
Chemotherapy	2 (2.9)	2 (8.7)	1 (12.5)
Radiotherapy	3 (4.3)	5 (21.7)	5 (62.5)

MC: medullary carcinoma; PDC: poorly differentiated carcinoma; AC: anaplastic carcinoma.
